# Temporal development and collapse of an Arctic plant-pollinator network

**DOI:** 10.1186/1472-6785-9-24

**Published:** 2009-12-04

**Authors:** Clementine Pradal, Jens M Olesen, Carsten Wiuf

**Affiliations:** 1 Ecole Centrale Paris, Grande Voie des Vignes, F-92 295 Chatenay-Malabry Cedex, France; 2 Bioinformatics Research Centre, Aarhus University, C. F. Mollers Alle 8, Building 1110, DK-8000 Aarhus C, Denmark; 3 Department of Biological Sciences, Aarhus University, Ny Munkegade, Building 1540, DK-8000 Aarhus C, Denmark

## Abstract

**Background:**

The temporal dynamics and formation of plant-pollinator networks are difficult to study as it requires detailed observations of how the networks change over time. Understanding the temporal dynamics might provide insight into sustainability and robustness of the networks and how they react to environmental changes, such as global warming. Here we study an Arctic plant-pollinator network in two consecutive years using a simple mathematical model and describe the temporal dynamics (daily assembly and disassembly of links) by random mechanisms.

**Results:**

We develop a mathematical model with parameters governed by the probabilities for entering, leaving and making connections in the network and demonstrate that A. The dynamics is described by very similar parameters in both years despite a strong turnover in the composition of the pollinator community and different climate conditions, B. There is a drastic change in the temporal behaviour a few days before the end of the season in both years. This change leads to the collapse of the network and does not correlate with weather parameters, C. We estimate that the number of available pollinator species is about 80 species of which 75-80% are observed in each year, D. The network does not reach an equilibrium state (as defined by our model) before the collapse set in and the season is over.

**Conclusion:**

We have shown that the temporal dynamics of an Arctic plant-pollinator network can be described by a simple mathematical model and that the model allows us to draw biologically interesting conclusions. Our model makes it possible to investigate how the network topology changes with changes in parameter values and might provide means to study the effect of climate on plant-pollinator networks.

## Background

The structure of plant-pollinator networks and other ecosystems has been described through features reflecting their topology and complex organization [1-3]. Also, aspects of the network dynamics have been studied, mainly from the perspective of understanding the principles that rule the number and choice of connections made by species in the network. One prominent example is the model of preferential attachment [4,5], where new species entering the network tend to link to species already well-connected. Such models have been fitted to empirical pollination networks, see e.g. [6].

One key aspect of plant-pollinator networks remains little explored, namely the temporal dynamics. The temporal dynamics describes how a network is formed and modified over time, its sustainability and robustness and the mutual activities of plants and pollinators over time. Plant-pollinator networks are susceptible to environmental changes such as global warming [7-9]; hence insight into the temporal dynamics might provide valuable information about the impact of environmental changes. This is, in particular, of relevance in the Arctic where global warming is expected to have the most severe effects [10]. In the present study, we define temporal dynamics as the development of the number of species and links in the network for a given period of time, typically a season. Here we use *season *in the sense of the network's *activity period*, i.e. the time when both flowering plants and pollinators are present. A season begins with the observation of a first link between an animal and a plant. From this starting point the network typically experiences a phase of growth where many species enter the network and with an increase in the number of connections until a slackening of the expansion appears. A collapse of the network follows with a reduction in the numbers of the links and species previously involved in the structure. In regions where climate conditions are favourable, the network can exist all year round [2] with fluctuations in its size throughout the year and without a complete cessation of the activity. In regions with stringent climate conditions the length of the season can be reduced to a few weeks or months because of the presence of snow and low temperatures.

A few studies explore the temporal dynamics of pollination networks [3,6,11-14]. Common to most of these studies is that they highlight the strong temporal dynamics of several variables, e.g. species number, species linkage level (number of links of a species to other species), total number of links in the network, network connectance and nestedness. However, these studies are based on bi-weekly, monthly or annual collection of data and comparisons, and do not propose models to describe and explain the observations, let alone the dynamics, but mainly base analysis on descriptive statistics.

Only [6] discusses the day-to-day dynamics and compares the fit of a (truncated) power-law and an exponential distribution to the plant and pollinator linkage levels. They use arctic plant-pollinator data collected over two consecutive seasons near Zackenberg Meteorological Station (ZMS) in Greenland. However, neither distribution is able to explain the daily development (assembly and disassembly) of links and species in the network. Here we develop a mathematical model to account for the day-to-day dynamics of the network data in [6]. The completeness of the data enables us to follow and describe the network development using simple mathematical tools and to provide a model that reproduces aspects of the dynamics. By comparing the two seasons we assess the stability of the model parameters over the years, the influence of weather on the development of the network and draw biological consequences of the model.

## Results and Discussion

### The start and end of the season

**Figure 1:**
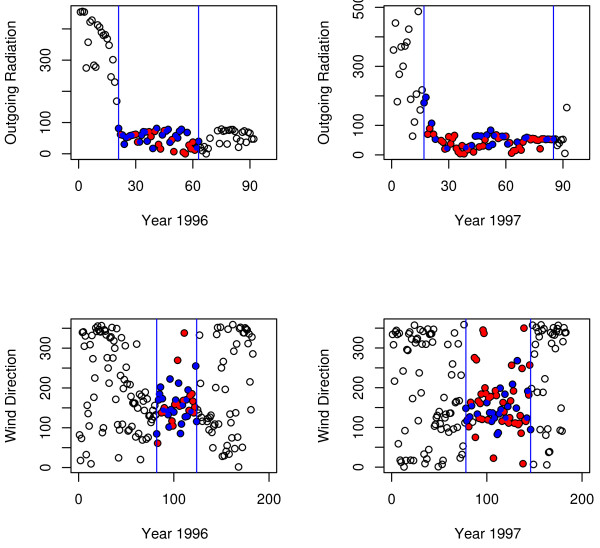
**The start and end of the season**. The figure shows the amount of outgoing radiation at 12 noon from June to August in 1996 and 1997 and the wind direction at 12 noon from April to September, also in 1996 and 1997. The start of the season correlates strongly with reduced level of outgoing radiation, whereas both the start and the end of the season correlate mildly with wind direction (0° -360°). The season is marked with two vertical blue lines; good days (days of observation) are blue and bad days (days where observations were not done due to bad weather conditions) are red. In the case of outgoing radiation (top two figures) days are counted from June 1st (i.e. June 1st = Day 0) and in the case of wind direction days are counted from April 1st (i.e. April 1st = Day 0). Note that 0° and 360° represent the same direction.

In the Arctic the start of the flowering season correlates strongly with snow melt and a corresponding rapid large change in net and outgoing radiation [9,10,15]. This is also the case for our data sets, see Figure 1. In contrast, the end of the season is not as strongly correlated with changes in net and outgoing radiation, or other climatic parameters [9]. However, the wind direction does show some correlation with both the start and the end of the season, see Figure 1. Further, in our case, we note that weather parameters generally differ between years (Table 1).

**Table 1:** Summary of weather parameters

Year	Month	Temp (C)	Net (W/m^2^)	Wind (m/s)
1996	June	1.90 (2.76)	106.58 (145.84)	1.39 (0.93)
	July	5.84 (3.58)	137.10 (149.48)	2.28 (1.38)
	August	4.40 (3.20)	68.78 (106.68)	2.50 (1.89)
1997	June	2.23 (2.88)	80.48 (105.61)	2.06 (1.65)
	July	3.72 (2.52)	123.35 (126.24)	2.40 (1.86)
	August	5.05 (3.69)	70.74 (111.34)	2.45 (1.74)

Shown are mean values with standard deviations in parenthesis of air temperature (Temp), net radiation (Net) and wind velocity (Wind) for the three summer months in 1996 and 1997.

### The collapse of the network

**Figure 2:**
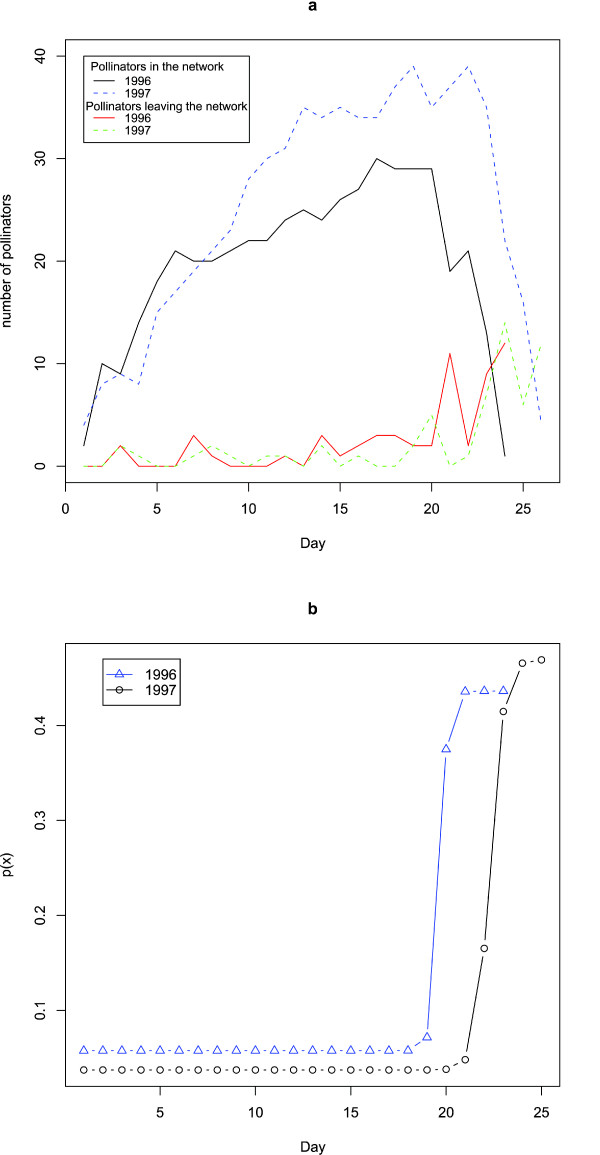
**Number of pollinators**. a) Evolution of the number of pollinators in the network during the season. For both years, the number of pollinators increases until a few days before the end of the season where it collapses suddenly. At the same time, the number of pollinators leaving the network peaks. b) Shown is the estimated sigmoid shape of the parameter *p*(*x*) (see Methods, Analysis and modelling) for the removal of insects in 1996 and 1997. 'Day' is the good days.

Figure 2 shows that the number of pollinators decreases drastically a few days before the end of the season. This is the case for both years and indicates the beginning of the end of the season. Though the end of the season is not characterized by marked changes in weather conditions (see section above), the collapse of the network could still be related to (minor) changes or fluctuations in weather conditions that cause pollinators to disappear and/or reduce activity or plants to end flowering; see e.g. [10,16] where activity levels of Alpine insects are discussed in relation to varying weather conditions. To test this hypothesis we performed a linear regression of the number of pollinators leaving the network on the ZMS climatic variables from the same day (or the day before; we did both analyses); the analyses showed that the hypothesis is not supported. Generally we overestimate the number of leaving pollinators in the beginning of the season and underestimate the number at the end of the season (Additional file 1). It indicates that other factors than the available climatic variables affect the end of the season.

In contrast, we found that the disappearance of pollinators from the network is highly correlated with the disappearance of plants from the network (linear regression: *r*^2 ^= 0.8 for 1996, and *r*^2 ^= 0.7 for 1997). Since we only know the time span a plant is visited and not for how long it is flowering, we cannot say whether links disappear A) because plants stop flowering or B) because insects disappear for other reasons. Some of the plants that stay in the network longest also enter the network very early in the season (see Additional files 2 and 3). Based on the available data, it is therefore difficult to distinguish between A and B. However, we note that the collapse of the network follows immediately after the temperature has peaked: In 1996, the temperature rises above 15°C for several days and in 1997, the temperature rises above 20°C in a single day (Additional file 4). It is plausible that flowering of plants adapted to cold climate could be affected by high temperatures [17].

When interpreting the results it should be kept in mind that weather conditions to some extent could be local and heterogeneous; e.g. variations in the orientation of the ground level towards the sun could impose restrictions on the hours of sun, wind and the snow coverage of plants compared to ZMS. Heterogeneous snow distribution has previsouly been reported to influence e.g. alpine ecosystems [10,16].

### Simple distributions describe the temporal development

Our main focus is to describe the temporal dynamics of the network with simple probability distributions and compare the results between the two years. The composition of the plant community is the same for the two years; in total 31 flowering plants comprising the same species each year; see [6] and Additional file 2. Therefore we consider the plants as fixed and study the network from the view of the pollinators. In particular, we are interested in the following features:

• How many pollinators enter the network daily?

• How many pollinators leave the network daily?

• How many links do pollinators get when they first enter the network?

• How many links do pollinators gain daily while they are in the network?

• How many links do pollinators loose daily while they are in the network?

We use the answers to the above questions as a scaffold to develop a model of network assembly and disassembly. The daily arrival of pollinators can be described by a Poisson distribution (Table 2). The empirical graph of pollinators leaving the network shows a drastic change a few days before the end of both seasons (Figure 2, as discussed above). To accommodate this we fitted a binomial distribution to the number of leaving pollinators assuming a gradual sigmoid change in the parameter (see Table 2 and Methods, Analysis and modelling). Since the climatic parameters did not explain species disappearance we did not use these parameters for modelling.

**Table 2:** Model summary

	Parameter	1996	1997	1996&1997	LTR
Arrival of insects Poisson	*λ*	2.417 (0.317)	2.423 (0.305)	2.420 (0.220)	Yes
	*p*	0.094	0.15	0.28	0.99

Links for new insects Modified geometric	*r*_1_	0.414 (0.065)	0.429 (0.062)	0.421 (0.045)	Yes
	*r*	0.351 (0.049)	0.356 (0.048)	0.354 (0.034)	
	*p*	0.65	0.37	0.55	0.98

Death of insects Sigmoid binomial	*α*	0.058 (0.012)	0.037 (0.008)	0.046 (0.007)	Yes
	*β*	0.436 (0.071)	0.469 (0.143)	0.446 (0.063)	
	*H*	4.901 (4.942)	2.797 (3.688)	3.847 (3.984)	
	*T*	19.66 (0.362)	22.31 (0.747)		
	*p*	0.41	0.51	0.75	0.57

Addition of links Geometric	*q*	0.752 (0.019)	0.836 (0.015)	0.799 (0.012)	No
	*p*	0.13	0.078	0.031	*<*10^-3^

Removal of links Sigmoid binomial	*γ*	0.006 (0.002)	0.006 (0.002)	0.008 (0.002)	No
	*δ*	0.187 (0.021)	0.401 (0.036)	0.271 (0.020)	
	*h*	18.28 (9.497)	40.12 (-)*	48.68 (-)*	
	*t*	19.06 (0.031)	22.55 (-)*		
	*p*	0.15	0.73	*<*10^-3^	*<*10^-3^

Shown are the fitted parameters for the two years; estimated for each year separately and jointly (except *T *and *t*) with standard deviations in parenthesis. The p-values in the 3rd, 4th and 5th columns are goodness-of-fit probabilities based on the chi-square test; the p-values in the 6th column are test probabilities for the LRT (Yes/No indicates whether the LRT is accepted or not). Even when the LRT is not accepted the parameters are of similar magnitude in the two years. The distributions are explained in Methods, Analysis and modelling. Standard deviations marked with * could not be estimated reliably.

When pollinators first enter the network, many have few links while fewer have many links; 57% (resp. 63%) of the pollinators enter the network with one or two links and 12% (resp. 13%) have six or more links in 1996 (resp. 1997). We used a modified geometric distribution that allows the ratio of insects with one link to insects with two or more links to be higher compared to a pure geometric distribution (see details in Methods, Analysis and modelling).

**Figure 3:**
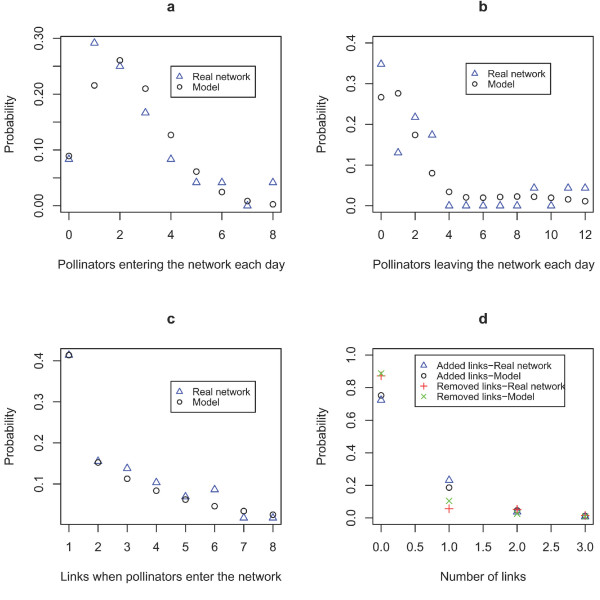
**Empirical and fitted distributions, 1996**. Dynamic features of the 1996 network and the associated distributions. a) Number of pollinators entering the network each day fitted to a Poisson distribution, b) Number of pollinators leaving the network each day fitted to a binomial distribution with sigmoid-shaped parameter, c) Number of links assigned to pollinators when they enter the network. Here fitted to a modified geometric distribution, d) Number of links added or removed each day from pollinators in the network. The model is a geometric distribution for the added links and a binomial distribution with a sigmoid-shaped parameter for the removed ones. See Additional file 5 for 1997.

Once in the network, pollinators can keep the same number of links - which is the most frequent situation; 65% (resp. 75%) of the cases in 1996 (resp. 1997) - get one or more additional links or loose one or more links each day. For the addition of links we fit a geometric distribution and for the loss of links a binomial distribution. The loss of links is more pronounced at the end of the season and we allowed a sigmoid form of the parameter again. All the computed parameters, and results of the tests for the chosen distributions are gathered in Table 2, Figure 3 (year 1996) and Additional file 5 (year 1997).

For the arrival of new pollinators and new links the fit is not improved significantly if a seasonal change in the parameters are allowed (results not shown) and there is not a visual indication of seasonal change. For the death of insects and removal of links the change in parameter happens around the same time (the parameters *T *and *t *are very similar, Table 2) and a model assuming *T *= *t *fits the data (results not shown). We also note that the change in parameter values over the season is very sharp and appears to happen within a day or two (Figure 2).

### Stability in the parameters over the two years

An important observation is that the estimated model parameters are similar for the two years, which indicates stability in the network dynamics. This hypothesis was validated for the arrival and leave of pollinators and for the number of links when they enter the network (Table 2). However the evolution of the number of links (addition or removal of links for pollinators while in the network) cannot be described by joined parameters. Despite of this, the parameters are still of the same order of magnitude. (In the remaining of the paper, the 'joined model' refers to a model which uses joined parameters whenever possible.)

While the plant community is the same for the two years, one fifth of the pollinator species and two thirds of all links are only observed in one of the two years; see [6] and Additional file 3. It is therefore interesting to see that the fitted distributions for the evolution of the number of pollinators and links through the season are identical for the two years with similar parameters. For other networks it has already been reported that overall network properties such as connectance or nestedness are conserved over the years despite the turnover in pollinator species and links [3,18]. Our findings reinforce this observation at the more detailed level, the level of the dynamical assembly and disassembly.

### Simulations

Based on the above results we implemented a computer program reproducing the arrival/leaving of pollinators and the evolution of their number of links with plants during their stay in the network (see Methods, Simulation). Addition of links is assumed to be independent of the pollinator's present linkage level. Also each link is removed with a probability independent of the linkage level. When pollinators are removed from the network they are chosen with probability inversely proportional to their linkage level. This provides a better fit to the observed networks than removing pollinators randomly and might reflect sampling properties; i.e. a pollinator with many links might have higher chance of being observed over consequetive days than a pollinator with few links.

We simulated 50 networks using the estimated parameters and compared the results to the observed data. Table 3 shows the simulated results and the comparisons; the observed data fits nicely to the model in that the observed data are within the 95%-confidence limits (the mean ± two times the standard deviation) obtained by simulation (Table 3).

**Table 3:** Validation of the model

	1996	1997
Maximum number of links	163 (29.5) 200	164 (35.8) 190
Total number of insects	57.7 (6.80) 61	64.6 (9.36) 64
Total number of interactions	277 (36.0) 286	266 (48.4) 268
Maximum number of insects	30.8 (5.29) 30	34.0 (6.00) 39
Connectance	0.155 (0.012) 0.15	0.133 (0.014) 0.14
Pollinator average linkage level	4.80 (0.381) 4.7	4.11 (0.421) 4.2
Distribution of		
Number of links when at maximum	35	48
Phenophase	50	38
Plants per pollinator	44	44

For each year and summary statistic, the mean and standard deviation (in parenthesis) are shown, based on 50 simulations. For each summary statistic, the second row shows the observed value. We used the parameters of the joined model. Connectance is defined as the number of observed links divided by the total number of potential links between all species of plants and animals. Bottom three rows: For each simulation it is tested whether the simulated distribution is similar to the observed distribution using Kolmogorov-Smirnov's test. Shown is the number of times (out of 50) the test gives a p-value larger than 0.05.

### Consequences of the model

The model assumes a Poisson number of arriving insects per day. This is compatible with a scenario where there are *N *available species and each insect has the same probability to visit and be observed at the study site; *λ *= *Np*_*i*_/*G*_*i *_where *p*_*i *_is the probability in year *i *and *G*_*i *_the number of good days (*λ *is assumed to be the same in both years). Under this scenario we expect *N*_1_*N*_2_/*N *= *N*_12 _insects to be observed both years. Here *N*_*i *_is the number of insects in year *i *and *N*_12 _the number of insects observed in both years. We find that *N *= *N*_1_*N*_2_/*N*_12 _= 61·64/49 = 79.7 pollinators (see Methods, Data sets), hence *p*_1 _= *N*_1_/*N *= 76.6% of the available pollinator species are observed in 1996, while *p*_2 _= 80.3% are observed in 1997. Solving *λ *= *Np*_*i*_/*G*_*i*_, gives *p*_1 _= 72.9% and *p*_2 _= 79.0% which are close to 76.6% and 80.3%, respectively, but derived through the Poisson model. The per day probability is independent of the year (since *λ *is the same), *p*_*i*_/*G*_*i *_= *λ*/*N *= 0.03.

If we assume that the parameters are fixed at the values they attain in the beginning of the season and that the season in principle goes on for ever, the network will eventually reach an equilibrium where the average pollinator phenophase approaches a constant level. Using 1996 parameters, the equilibrium phenophase is approximately 17.2 days, because a pollinator stays a geometric (with probability *α *= 0.058, Table 2) number of days. However the equilibrium level is not reached for season lengths observed in the Arctic (Table 4). Also the average number of pollinators in the network eventually reaches a constant level which is balanced by the arrival of new insects and the departure of insects already in the network. Our simulation shows that the real network is far from equilibrium and in the process of being built up when the collapse of the network appears (Table 4).

**Table 4:** Prolonging length of the season

Length of the season	Maximum number of links	Total number of insects	Average phenophase	Total number of links	Maximum number of insects	Pollinator average linkage level
24	200	61	7.89	286	30	4.7
30	230 (42.3)	74.1 (8.49)	8.72 (7.85)	385 (55.9)	37.0 (6.25)	5.19 (0.40)
40	312 (47.0)	95.3 (9.09)	10.7 (10.5)	552 (60.6)	41.7 (6.18)	5.79 (0.36)
60	475 (64.7)	147 (11.9)	13.6 (15)	992 (101)	49.1 (6.85)	6.73 (0.37)
80	625 (77.7)	193 (13.7)	15.9 (18.8)	1448 (138)	54.5 (6.12)	7.50 (0.40)

Shown are mean values with standard deviations in parenthesis based on 50 simulated networks. We used 1996 joined parameters (see Table 2). The length of the season is the number of good days only and the first row shows the observed data for the 1996 network. The 6th column shows the maximum number of insects present in a single day.

The model stipulates randomness in the development of the network. The linkage level of a pollinator is described by a sum of geometric (new links) and binomial (removal of links) variables. As shown in [6] the linkage level is far from a power-law and closer to an exponential (or geometric) distribution. Also a truncated power-law distribution (a power law restricted to a certain range defined by a cut-off) is fitted to the data in [6], but the cut-off is here difficult to reconcile with biological interpretation (see [19] for a discussion of power-laws in biology and [20] for a discussion and review of cut-offs/characteristic scales in ecology).

## Conclusion

### General remarks

Our study highlights various interesting points. Based on our model, we have demonstrated that the plant-pollinator network shows strong dynamic stability over the two years; i.e. the dynamic features of the network are highly conserved from one year to the next. The length of the season, temperature and other weather parameters differ (Table 1) and also the visiting pollinators are not the same. Despite this we find that the development of the network could be described by very similar parameters in 1996 and 1997. We described the number of links attached to a new insect by a modified geometric distribution. This distribution does not have the characteristic power-law shape that has been reported for other types of network and is in concordance with a previous analysis of the same data [6]. In addition, our model has distinctive random features; e.g. the number of new species per day is independent of the number already present and the number of insects being removed from the network daily is binomial such that each insect has the same probability of being removed.

Studies about the impact of habitat loss on pollination networks highlight the existence of a habitat destruction threshold at which both plants and pollinators disappear suddenly, leading to the collapse of the network [21]. The transition from maintenance to destruction of the network is very sharp leading to the conclusion that the network's fate might change by a slight modification of the parameter controlling the transition - almost like a phase transition. We observe a similar collapse of the network with parameters changing many folds within a few days. In our case, it could be that a sudden short rise in temperature causes plants to stop flowering [17]. Alternatively, it could be that night frost towards the end of the season creates north-facing snow patches that persist during day time, thereby reducing the resources available for the insects and making it more difficult for them to recover and regain activity. However, to test this hypothesis we need data on frost (and snow coverage) in the study site, which are not available. Finally, we demonstrated by simulation that the arrival and departure of insects in network has not reached equilibrium when the collapse of the network appears at the end of the season and we estimated that the number of available species in the area is about 80, of which 75%-80% are observed.

Climate change might have an impact on plant-pollinator networks [7,8,22-25]. For example an increase in the average temperature is likely to increase the length of the season and change the conditions for the existence and maintenance of the network. The effect of temperature rise is not well-understood, though some evidence is available. In [26], it is argued that experimental warming does not alter the length of the flowering season, whereas [27] and [28] (a study on butterflies) provide evidence that the adult life cycle of insects is unchanged with increasing temperature. However, the availability of pollinator species or plant species might change as the length of the season changes, as well as when the species are present [7]. To study consequences of climate changes, observations over several years would help us to relate the parameters to climatic variables; however we might still need to impose more assumptions on the model, e.g. for how long are plants flowering, to what extent do pollinators overlap with their plants etc.

### Remarks on and limitations of the model

We consider our model a first step in modelling the temporal dynamics of plant-pollinator networks, a complex process of network formation. As discussed in the sections above, species-specific information about plant and pollinators is not included in the model. This information includes species identity, species abundance, known species-specific interactions and weather-related parameters, such as temperature thresholds for when species are active and when they rest. In our case, species identity (Additional files 2 and 3) and the specific plant-pollinator interactions [6] are available. All this information potentially influence the dynamics of the network, e.g. when and which connections are made in the network [10,16,29]. However, it is not straightforward how to include such elaborate information in the model and the analysis we have proposed. Our model is based on network data gathered over two years and we have demonstrated that many aspects of the dynamics can be accounted for using simple mathematical tools. With detailed observations over several years we might be able to provide models that can account for further aspects of the temporal dynamics and include more detailed describtions of plants, pollinators and their characteristics.

## Methods

### Data sets

Our study is based on the plant-pollinator network of Zackenberg in Greenland [6]. Data were collected in 1996 and 1997 and include two full seasons from the first observation of insects visiting flowering plants to the last observation. In 1996 the season is 43 days from June 21 to August 2, while in 1997 it is 69 days from June 17 to August 24. However, bad weather reduced the number of days of observation to 24 and 26, respectively. Days of bad weather conditions were determined on site by the scientists involved in collecting the data. In the following 'good days' refers to days of observation and 'bad days' to days where observation was not possible. The study site is 500 m × 500 m.

For each day of observation we have a matrix describing the existing links between plants and pollinators present in the network at this day (Additional files 2 and 3 show summaries of the data: plant and pollinator phenology, respectively). An animal is linked to a plant species if the animal visits the flower of the plant. A link between a plant and a pollinator is supposed to exist from the first day when the link is observed to the last day when it is observed, irrespectively whether it is observed in all the intermediate days [6]. In total, the 1996 (1997) network includes 31 (31) plant species, 61 (64) pollinators and 286 (268) different links. The plant species are the same both years. In contrast 49 pollinators are observed both years with 79 different pollinators in total. The sampling effort was assessed in [6] and found to be good. Climatic data were collected from ZMS including temperature, amount of radiations (in, out and net radiation), and wind speed and direction http://www.zackenberg.dk; see Table 1. We obtained full information for the 1996 season, while in the 1997 season two days (June 24 and 25) were missing.

### Analysis and modelling

We used the following:

• Each day a Poisson number *Po*(*λ*) of insects enter the network

• Each day a binomial number *Bi*(*N*(*x *- 1), *p*(*x*)) of insects leave the network, where *x *is time, *N*(*x *- 1) is the number of insects present in the network on the previous day and

Early in the season *p*(*x*) is *α*, and late *β*. At time *T*, *p*(*T*) = (*α *+ *β*)/2; *H *controls how rapid the shift from *α *to *β *is. We assume at least one insect remains in the network each day

• When an insect enters the network, it is assigned a number of links *k *according to a modified geometric distribution:

- The probability of *k *= 1 link is *r*_1_

-The probability of *k >*1 links is (1 - *r*_1_)*r*(1 - *r*)^*k*-2^

• Each day, each pollinator in the network receives new links according to a geometric distribution, *Geo*(*q*)

• Each day, each pollinator in the network looses a binomial number of links *Bi*(*N*_*i*_(*x *- 1), *q*(*x*)), where *x *is time, *N*_*i*_(*x *- 1) is the number of links of pollinator *i *on the previous day and

At time *t*, *q*(*t*) = (*γ *+ *δ*)/2, where *γ *is the value of *q*(*x*) early in the season and *δ *the value late. We assume at least one link remains in the network each day.

Parameters were estimated using maximum likelihood, separately for each year and for the two years jointly. To test the validity of our models chi-square goodness-of-fit tests were used with simulated p-values because of small expected numbers. We used the Likelihood Ratio Test (LTR) to test whether parameters for the two seasons could be assumed identical. The analysis and tests were performed in R http://www.R-project.org. Logistic regression analysis on meteorological data was done using the package nnet in R.

### Simulation

To simulate a network from our model we wrote a program in R based on the distributions described in the previous section. We used estimated parameters, but the program allows arbitrary parameters as input, including season length (number of good days, i.e. days of observation). A simulation goes through the following steps:

• The length of the season is fixed from the beginning

• For each day, the number of new pollinators is defined according to a Poisson law. The sum of new pollinators over the season defines the pollinator list. The date of arrival is attributed to each pollinator

• For each day of the season, a number of links (modified geometric distribution) is attributed to the pollinators entering the network on this day. Links can be added to pollinators already in the network (geometric distribution) or deleted (binomial distribution)

• For each day of the season, the number of pollinators leaving the network is determined according to a binomial law. The leaving pollinators are then chosen with a probability inversely proportional to their linkage level (i.e. probability 1/*k*, where *k *is the number of links of the pollinator).

We simulated 50 networks and compared various features to the real networks. For each feature, we calculated the Z-score *Z *= (*X *- *μ*)/*σ*, where *X *is the value in the real network, *μ *the mean of the simulations and *s *the standard deviation of the simulations; the distribution of simulated values are approximately normal (results not shown). To compare distributions, the function ks.boot from the package Matching in R which performs a Kolmogorov-Smirnov test with bootstrapping, was used.

## Authors' contributions

All authors were involved in designing the study; CP did the analyses and drafted the paper; CP and CW finalized the paper with input from JMO; JMO provided the network data. All authors read and approved the final manuscript.

## Supplementary Material

Additional file 1 **Prediction of collapse**. Linear regression of climatic parameters on the number of pollinators leaving the network. Here we use temperature, wind and net radiation. Simple combinations of climatic parameters cannot predict the collapse and end of the season observed as a decline in the number of pollinators. 'Day' is the good days. Click here for file

Additional file 2 Plant species present and their phenology, 1996 and 1997. Click here for file

Additional file 3 Pollinator species present and their phenology, 1996 and 1997. Click here for file

Additional file 4 **Temperature through the season**. The figure shows the temperature at 12 noon from June to August in 1996 and 1997. The season is marked with two vertical blue lines; good days are blue, bad days are red. Days are counted from June 1st, i.e. June 1st = Day 0. Click here for file

Additional file 5 **Empirical and fitted distributions, 1997**. Dynamic features of the 1997 network and associated models. a) Number of pollinators entering the network each day fitted to a Poisson distribution, b) Number of pollinators leaving the network each day fitted to a binomial distribution with sigmoid-shaped parameter, c) Number of links assigned to pollinators when they enter the network. Here fitted to a modified geometric distribution, d) Number of links added or removed each day from pollinators in the network. The model is a geometric distribution for the added links and a binomial distribution with a sigmoid-shaped parameter for the removed ones. Click here for file
