# Supplementary figures and images for: Temporal development and collapse of an Arctic plant-pollinator network
Source: BMC Ecol. 2009 Dec 4;9:24. doi: 10.1186/1472-6785-9-24 (PMC2800837; doi:10.1186/1472-6785-9-24)

**1996**

Number of insects leaving the network

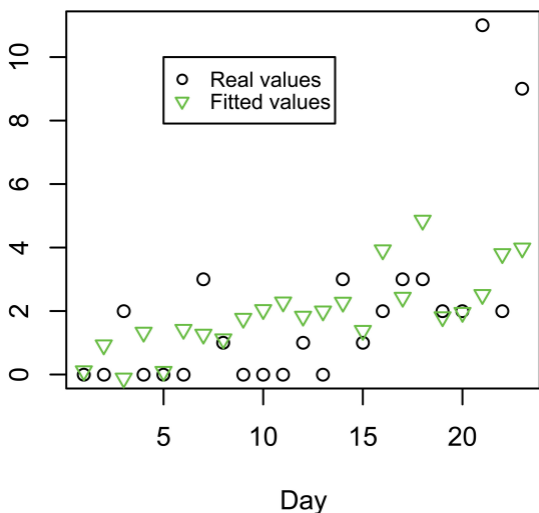

**1997**

Number of pollinators leaving the network

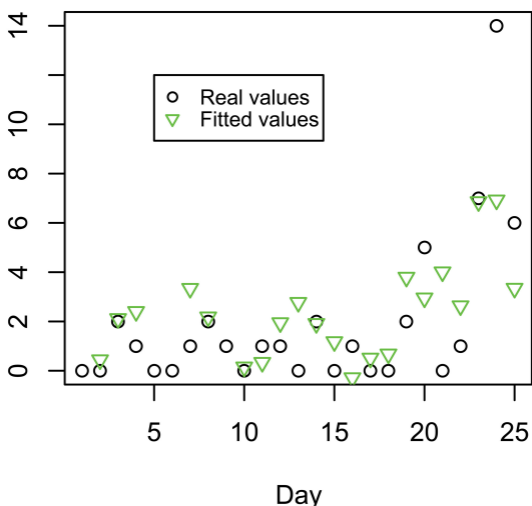

Supplement: Additional file 1 — Prediction of collapse. Linear regression of climatic parameters on the number of pollinators leaving the network. Here we use temperature, wind and net radiation. Simple combinations of climatic parameters cannot predict the collapse and end of the season observed as a decline in the number of pollinators. 'Day' is the good days. [file 1472-6785-9-24-S1.PDF]

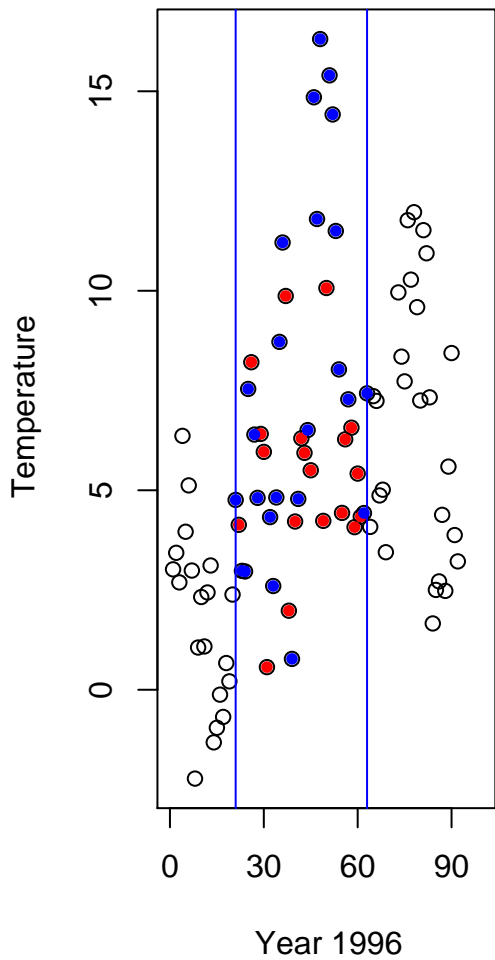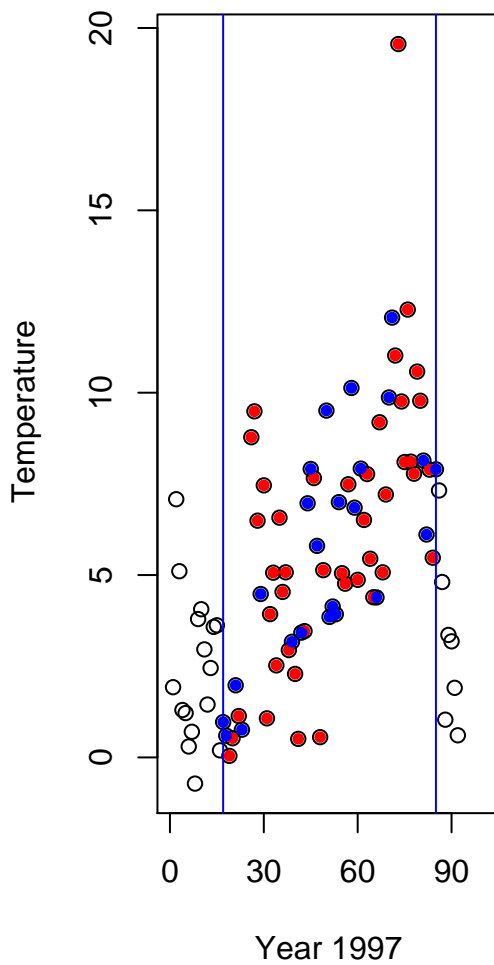

Supplement: Additional file 4 — Temperature through the season. The figure shows the temperature at 12 noon from June to August in 1996 and 1997. The season is marked with two vertical blue lines; good days are blue, bad days are red. Days are counted from June 1st, i.e. June 1st = Day 0. [file 1472-6785-9-24-S4.PDF]

**a**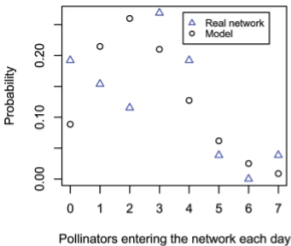**b**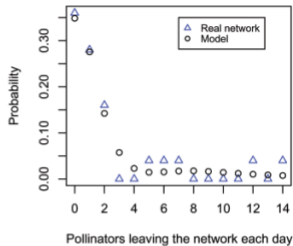**c**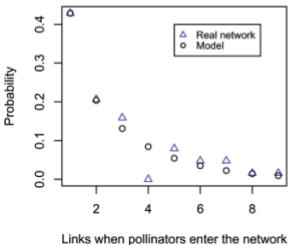**d**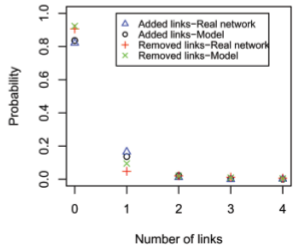

Supplement: Additional file 5 — Empirical and fitted distributions, 1997. Dynamic features of the 1997 network and associated models. a) Number of pollinators entering the network each day fitted to a Poisson distribution, b) Number of pollinators leaving the network each day fitted to a binomial distribution with sigmoid-shaped parameter, c) Number of links assigned to pollinators when they enter the network. Here fitted to a modified geometric distribution, d) Number of links added or removed each day from pollinators in the network. The model is a geometric distribution for the added links and a binomial distribution with a sigmoid-shaped parameter for the removed ones. [file 1472-6785-9-24-S5.PDF]
