# Supplementary material for: Temporal development and collapse of an Arctic plant-pollinator network
Source: BMC Ecol. 2009 Dec 4;9:24. doi: 10.1186/1472-6785-9-24 (PMC2800837; doi:10.1186/1472-6785-9-24)
Supplement: Additional file 2 — Plant species present and their phenology, 1996 and 1997. [file 1472-6785-9-24-S2.PDF]

1 y: day 1 = 21 June<br>2 y: day 1 = 17 June<br>Pheno lgth = phenophase length	presence	start date	end date	start	end	pheno lgth	presence	start date	end date	start	end	pheno lgth
Arenaria pseudofrigida	1	03-jul	22-jul	13	32	20	1	14-jul	10-aug	28	55	28
Armeria scabra	1	05-jul	23-jul	15	33	19	1	29-jul	21-aug	43	66	24
Arnica angustifolia	1	17-jul	01-aug	27	42	16	1	10-aug	21-aug	55	66	12
Cassiope tetragona	1	02-jul	23-jul	12	33	22	1	23-jun	10-aug	7	55	49
Cerastium arcticum	1	23-jun	01-aug	3	42	40	1	18-jun	24-aug	2	69	68
Chamaenerion latifolium	1	11-jul	01-aug	21	42	22	1	10-aug	10-aug	55	55	1
Cochlearia groenlandica	1	22-jul	27-jul	32	37	6	1	5-aug	10-aug	50	55	6
Draba arctica	1	02-jul	02-jul	12	12	1	1	24-jun	17-jul	8	31	24
Draba lactea	1	22-jul	23-jul	32	33	2	1	9-aug	10-aug	54	55	2
Dryas octopetala	1	21-jun	27-jul	1	37	37	1	17-jun	20-aug	1	55	55
Erigeron compositus	1	06-jul	23-jul	16	33	18	1	14-jul	14-jul	28	28	1
Lesquerella arctica	1	23-jun	23-jun	3	3	1	1	18-jun	24-jun	2	8	7
Melandrium triflorum	1	28-jun	01-aug	8	42	35	1	25-jun	21-aug	9	66	58
Papaver radicatum	1	23-jun	27-jul	3	37	35	1	18-jun	21-aug	2	66	65
Pedicularis flammea	1	27-jun	09-jul	7	19	13	1	22-jul	22-jul	36	36	1
Pedicularis hirsuta	1	25-jun	22-jul	5	32	28	1	15-jul	28-jul	29	42	14
Polygonum viviparum	1	22-jul	02-aug	32	43	12	1	20-jul	24-aug	34	69	36
Potentilla hyparctica	1	04-jul	23-jul	14	33	20	1	18-jun	10-aug	2	55	54
Potentilla rubricaulis	1	27-jun	23-jul	7	33	27	1	18-jun	10-aug	2	55	54
Ranunculus sulphureus	1	18-jul	24-jul	28	34	7	1	17-jul	10-aug	31	55	25
Rhododendron lapponicum	1	21-jun	02-jul	1	12	12	1	25-jun	14-jul	9	28	20
Salix arctica	1	23-jun	27-jul	3	37	35	1	17-jun	10-aug	1	55	55
Saxifraga caespitosa	1	21-jul	01-aug	31	42	12	1	28-jul	20-aug	42	65	24
Saxifraga cernua	1	23-jul	01-aug	33	42	10	1	20-jul	21-aug	34	66	33
Saxifraga hirculus	1	01-aug	02-aug	42	43	2	1	23-jul	21-aug	37	66	30
Saxifraga nivalis	1	02-jul	27-jul	12	37	26	1	14-jul	10-aug	28	55	28
Saxifraga oppositifolia	1	23-jun	23-jul	3	33	31	1	17-jun	20-aug	1	55	55
Silene acaulis	1	23-jun	01-aug	3	42	40	1	29-jun	24-aug	13	69	57
Stellaria longipes	1	22-jul	01-aug	32	42	11	1	10-aug	21-aug	55	66	12
Taraxacum phymatocarpum	1	27-jul	27-jul	37	37	1	1	24-jul	10-aug	38	55	18
Vaccinium uliginosum	1	03-jul	16-jul	13	26	14	1	25-jun	17-jul	9	31	23
TOTAL plant species	31						31					
