# Supplementary material for: Temporal development and collapse of an Arctic plant-pollinator network
Source: BMC Ecol. 2009 Dec 4;9:24. doi: 10.1186/1472-6785-9-24 (PMC2800837; doi:10.1186/1472-6785-9-24)
Supplement: Additional file 3 — Pollinator species present and their phenology, 1996 and 1997. [file 1472-6785-9-24-S3.PDF]

1996

1 y: day 1 = 21 June
Pheno lgth = phenophase length

	start date	end date	pheno lgth
A. nigripes	5	37	33
Parasyrphus tarsatus	5	12	8
L. brachytomus	7	46	40
Rhamphomyia filicauda	7	46	40
P. lundbecki	7	41	35
Phaonia bidentata	7	20	14
S. sanctipauli	7	37	31
Plebeius glandon spp. franklinii	7	7	1
Nysius groenlandicus	7	37	31
Genus sp.	7	7	1
Bombus hyperboreus, B. polaris	8	16	9
Aedes impiger	9	37	29
Paraphaenocladius spp.	9	37	29
Smittia spp.	9	38	30
Clossiana chariclea, C. ?	9	47	39
Colias hecla	9	41	33
Cricotopus sp.	11	25	15
Bradysia spp.	11	41	31
R. nigrita	11	46	36
D. segnis	11	35	25
Chironomus sp.	12	12	1
Eupeodes curtus	12	12	1
Spilogona almqvistii	12	46	35
Limnophyes spp.	16	35	20
Culicoides sp.	16	25	10
Chironomidae indet.	17	36	20
Brachypogon (subg. Isohelea) sp.	18	30	13
Phytomyza fuscula	19	31	13
Pseudosmittia sp.	23	46	24
Rheocricotopus sp.	23	37	15
S. micans	23	41	19
Drymeia groenlandica	25	25	1
S. dorsata	28	46	19
S. melanosoma	28	28	1
Scatopsciara sp.	30	37	8
Olethreutes inquitana	30	30	1
Stenomacrus sp. A	30	46	17
Megaselia sp.	31	31	1
Peleteria aenea	31	37	7
Sympistis lapponica	31	31	1
Lasiopiophila pilosa	32	36	5
Gonarcticus arcticus	32	32	1
S. denudata	32	46	15
S. extensa	32	46	15
Genus sp.	32	32	1
Aphidius? sp.	32	32	1
Genus sp.	32	32	1
Limnophora sinuata	35	46	12

Protophormia terraenovae	35	37	3
Orthocladius sp.	36	37	2
Scathophaga furcata	36	41	6
Genus sp.	36	36	1
S. obsoleta	37	37	1
S. tornensis	37	41	5
Lophosceles minimus	38	38	1
Platycheirus carinatus	41	41	1
Neoleria prominens	41	41	1
Stenomacrus sp. B	41	46	6
Stenomacrus sp. C	41	41	1
Genus sp.	41	46	6
S. tundrae	46	46	1

1997

2 y: day 1 = 17 June
Pheno lgth = phenophase length

	start date	end date	pheno lgth
Platycheirus carinatus	17-jun	20-aug	65
Platycheirus lundbecki	17-jun	21-aug	66
Smittia spp.	17-jun	21-aug	66
Bradysia spp.	17-jun	24-aug	69
Genus sp.	18-jun	18-jun	1
Olethreutes mengelana	18-jun	18-jun	1
Spilogona sanctipauli	18-jun	21-aug	65
Rhamphomyia filicauda	18-jun	24-aug	68
Genus sp.	21-jun	21-jun	1
Phaonia bidentata	21-jun	21-jun	1
Parasyrphus tarsatus	21-jun	21-aug	62
Procladius sp.	24-jun	25-jun	2
Bombus hyperboreus, B. polaris	24-jun	09-jul	16
Paraphaenocladius spp.	24-jun	28-jul	35
Aedes impiger	24-jun	29-jul	36
Drymeia segnis	24-jun	29-jul	36
Limnophyes spp.	24-jun	29-jul	36
Brachypogon (subg. Isohelea) sp.	24-jun	10-aug	48
Polia richardsoni	25-jun	10-aug	47
Zaphne frontata	25-jun	10-aug	47
Genus sp.	29-jun	29-jun	1
Olethreutes inquitana	29-jun	29-jun	1
Drymeia groenlandica	29-jun	22-jul	24
Eukiefferiella sp.	09-jul	20-jul	12
Orthocladiinae indet.	09-jul	09-aug	32
Limnophyes brachytomus	09-jul	21-aug	44
Spilogona almqvistii	09-jul	24-aug	47
Aedes nigripes	12-jul	10-aug	30
Rhamphomyia nigrita	12-jul	10-aug	30

Spilogona tornensis	12-jul	10-aug	30
Culicoides sp.	14-jul	14-jul	1
Colias hecla	14-jul	10-aug	28
Spilogona dorsata	14-jul	19-aug	37
Spilogona extensa	14-jul	20-aug	38
Cricotopus sp.	14-jul	21-aug	39
Spilogona deflorata	15-jul	15-jul	1
Delia echinata	15-jul	20-jul	6
Stenomacrus sp. A	15-jul	24-aug	41
Eupeodes nigroventris	17-jul	09-aug	24
Clossiana chariclea	17-jul	21-aug	36
Limnophora sinuata	20-jul	10-aug	22
Spilogona tundrae	20-jul	10-aug	22
Pseudosmittia sp.	20-jul	20-aug	32
Nysius groenlandicus	20-jul	21-aug	33
Eupeodes curtus	21-jul	20-aug	31
Spilogona micans	22-jul	21-aug	31
Spilogona malaisei	28-jul	28-jul	1
Lophosceles minimus	28-jul	21-aug	25
Spilogona denudata	28-jul	21-aug	25
Rheocricotopus sp.	29-jul	29-jul	1
Stenomacrus sp. B	29-jul	29-jul	1
Spilogona arcticola	29-jul	09-aug	12
Forcipomyia sp.	29-jul	10-aug	13
Entephria puncticeps	31-jul	09-aug	10
Plebeius glandon spp. franklinii	05-aug	05-aug	1
Sympistis zetterstedti	05-aug	20-aug	16
Genus sp.	09-aug	09-aug	1
Megaselia sp.	09-aug	09-aug	1
Spilogona melanosoma	09-aug	09-aug	1
Syngrapha parilis	09-aug	10-aug	2
Lasiopiophila pilosa	10-aug	10-aug	1
Neoleria prominens	10-aug	10-aug	1
Peleteria aenea	10-aug	21-aug	12
Scathophaga furcata	20-aug	20-aug	1
